# Isolation and Genome-Resolved Characterisation of Selected Indigenous Lactic Acid Bacteria from Traditional Fermented Dairy Products of Kazakhstan

**DOI:** 10.3390/microorganisms14102201

**Published:** 2026-10-01

**Authors:** Aidana Utebaeva, Eleonora Gabrilyants, Zhansaya Abish, Aigerim Ablash, Inga Ciprovica

**Affiliations:** 1 Textile and Food Engineering Higher School, Mukhtar Auezov South-Kazakhstan University, Tauke Khan Avenue 5, Shymkent 160012, Kazakhstan; gabrilyantse35@gmail.com (E.G.); abish.zhansaya95@mail.ru (Z.A.); aikosha_1999@inbox.ru (A.A.); 2 Food Institute, Faculty of Agriculture and Food Technology, Latvia University of Life Sciences and Technologies, LV-3001 Jelgava, Latvia; inga.ciprovica@lbtu.lv

**Keywords:** lactic acid bacteria, traditional dairy products, indigenous strains, whole-genome sequencing, *Leuconostoc mesenteroides*, *Lactococcus lactis*, *Lentilactobacillus* ssp.

## Abstract

Traditional fermented dairy products of Kazakhstan represent potential sources of indigenous lactic acid bacteria (LAB). However, the cultivable LAB associated with these products remain insufficiently characterised. This study aimed to isolate and taxonomically characterise cultivable LAB recovered from traditional dairy products of Kazakhstan using a culture-dependent workflow combined with MALDI-TOF MS, 16S rRNA gene sequencing, phylogenetic analysis, and whole-genome sequencing (WGS) of selected isolates. A total of 102 samples, including kumis, shubat, raw mare’s milk, raw camel’s milk, and freshly prepared soft camel milk cheese, were analysed. Samples were cultured on MRS agar under aerobic conditions at 30 °C for 48 h, and colonies were collected from randomly selected sectors of the plates and purified for further analysis, irrespective of their morphology. In total, 315 bacterial isolates were recovered, of which 79 were confirmed as LAB and retained for further taxonomic analysis. Eight LAB species belonging to four genera were identified, with *Leuconostoc mesenteroides* being predominant among the recovered LAB isolates (48/79, 60.8%), followed by *Lactococcus lactis* (19/79, 24.1%). *Lentilactobacillus hilgardii*, *Lentilactobacillus kefiri*, and *Lentilactobacillus diolivorans* were detected only among isolates recovered from fermented products, whereas *Lacticaseibacillus paracasei* was detected at low frequency. Five representative isolates, including both frequently and less frequently recovered species, were selected for WGS. WGS supported the taxonomic assignments and yielded draft genome assemblies ranging from 1.68 to 2.69 Mb, with GC contents of 34.8–43.1%. OrthoANI analysis further supported their species-level assignments and enabled subspecies-level resolution for selected isolates. Overall, the study establishes a culture-based collection of indigenous LAB recovered from traditional dairy products of Kazakhstan, representing the genera *Leuconostoc*, *Lactococcus*, *Lentilactobacillus*, and *Lacticaseibacillus*. The observed LAB species composition reflects the cultivable fraction recovered under the applied cultivation and colony-selection conditions and should not be interpreted as a comprehensive representation of LAB diversity in the investigated products. The resulting strain collection and genome sequences provide a basis for subsequent comparative genomic, physiological, and technological studies of selected indigenous LAB.

## 1. Introduction

Traditional fermented dairy products constitute stable, self-organising microbial ecosystems that develop through spontaneous fermentation and the long-term co-evolution of microorganisms with raw materials, environmental conditions, and local processing practices [1,2]. Unlike industrial products produced with standardised starter cultures, these systems are characterised by high microbial variability, complex microbial interactions, and the presence of autochthonous strains adapted to specific ecological niches [3,4].

In recent years, interest in fermented food systems has grown considerably due to their nutritional, technological, and functional importance. Lactic acid bacteria are regarded as key functional components of fermented dairy ecosystems because of their role in lactose fermentation, acidification, flavour formation, and the production of organic acids, aroma compounds, and antimicrobial metabolites [5,6,7,8,9]. Furthermore, certain LAB species exhibit probiotic properties and may contribute to human health through modulation of the gut microbiota [6,10].

Despite significant progress in the study of fermented food microbiomes, most investigations rely on 16S rRNA gene amplicon sequencing or metagenomic analysis. These methods provide valuable information regarding microbial community composition but do not permit the isolation of viable microorganisms or the direct assessment of their genomic and functional characteristics. Consequently, many potentially valuable strains remain insufficiently characterised at the culture and genomic levels.

Culture-dependent approaches, including the culturomics strategy, combined with MALDI-TOF MS and WGS, enable these limitations to be overcome, facilitating both the isolation of pure cultures and their highly accurate taxonomic and functional characterisation. However, the application of such integrative approaches to traditional dairy products of Central Asia remains extremely limited [11,12,13].

Among the traditional fermented dairy products of Kazakhstan, kumis and shubat are of particular interest. Kumis is a fermented beverage produced from mare’s milk through mixed lactic acid and alcoholic fermentation involving LAB and yeasts [13,14]. Shubat is manufactured from camel’s milk and is characterised primarily by lactic acid fermentation, resulting in a product with higher acidity, viscosity, and solids content than kumis [11,12]. Both products are traditionally prepared using back-slopping techniques, whereby a portion of a previously fermented batch serves as a starter culture for fresh milk. As a result, their microbiota develops through repeated microbial succession and adaptation to local environmental conditions. Previous studies have reported that the microbiota of kumis and shubat is dominated by members of the genera *Lactococcus*, *Leuconostoc*, *Lentilactobacillus*, *Streptococcus*, and various yeast species, although the relative abundance of these microorganisms varies depending on geographical location, season, fermentation practices, and raw milk composition [3,4,5,6,14,15]. These characteristics make traditional Kazakh fermented dairy products valuable reservoirs of indigenous microbial diversity.

Another challenge in the study of traditional fermented foods, including those of Kazakhstan, is the limited applicability of commercial microbial identification systems. Although MALDI-TOF MS has become a widely used tool for rapid bacterial identification, incomplete reference databases frequently reduce its accuracy when analysing indigenous microorganisms from traditional food ecosystems. Similarly, 16S rRNA gene sequencing may not provide sufficient resolution to discriminate among closely related species, particularly within taxonomically complex groups such as *Leuconostoc*, thereby limiting taxonomic precision [16,17,18,19,20].

In this regard, the use of whole-genome sequencing becomes particularly relevant, as it allows not only the clarification of the taxonomic status of isolates but also the assessment of their genomic characteristics, potential metabolic capabilities, and evolutionary relationships [21]. However, genomic data for cultivable LAB isolated from traditional dairy products of Kazakhstan remain scarce, creating a significant gap in our understanding of regional microbial diversity and its potential applications.

Therefore, comprehensive investigations integrating culture-dependent isolation, MALDI-TOF MS, 16S rRNA gene sequencing, phylogenetic analysis, and WGS are required to improve our understanding of indigenous LAB associated with traditional fermented dairy products. Such studies are essential for identifying native strains with potential technological value and for developing regionally adapted starter cultures and functional fermented foods. Accordingly, the aim of the present study was to comprehensively characterise the cultivable diversity of lactic acid bacteria isolated from traditional dairy products of Kazakhstan using a multi-phase approach combining culture-dependent methods, MALDI-TOF MS, 16S rRNA gene sequencing, phylogenetic analysis, and whole-genome sequencing of selected indigenous strains.

## 2. Materials and Methods

### 2.1. Sample Collection

A total of 102 samples of traditional dairy products used in this study were collected in December 2025 from farms and privately owned mare-breeding farms (POMB) located in the Turkestan Region of the Republic of Kazakhstan (Figure 1). The sample set comprised 31 samples of kumis (fermented mare’s milk), 16 samples of shubat (fermented camel’s milk), 20 samples of raw mare’s milk, 28 samples of raw camel’s milk, and 7 freshly prepared camel milk cheeses.

Raw camel’s milk was collected from clinically healthy one-humped and two-humped camels maintained on the same farms. Milk samples were obtained from animals at different stages of lactation, including early and mid-lactation, during routine milking procedures. Raw mare’s milk was collected from clinically healthy mares at approximately 2, 4, and 6 months of lactation.

Freshly prepared kumis and shubat at an early stage of fermentation were collected from the same farms immediately after production, before prolonged storage or over-fermentation, to ensure representative microbial communities. Samples of freshly prepared soft cheese made from camel milk were obtained from a previously established collection of artisanal camel milk cheeses produced in the Turkestan Region of Kazakhstan and described in an earlier study [22].

All samples were aseptically collected into sterile containers. Liquid samples were collected in sterile containers with a capacity of 50–100 mL, while cheese samples were placed in sterile containers suitable for solid food samples. The samples were immediately stored at 4 ± 1 °C, transported to the laboratory under refrigerated conditions, and analysed within 24 h of collection.

### 2.2. Isolation and Cultivation of Lactic Acid Bacteria

To isolate lactic acid bacteria, samples were subjected to a tenfold serial dilution (10^−1^–10^−6^) in sterile saline solution (0.85% NaCl). From the appropriate dilutions (depending on the product type), 100 µL of the suspension was inoculated onto pH 6.5 MRS agar (de Man, Rogosa, and Sharpe, Condalab, Madrid, Spain, Cat No. 104300) using the surface plating method [23].

The inoculated plates were incubated aerobically at 30 °C for 48 h in a dry-air incubator (TV-80-1, Kasimov Instrument Plant, Kasimov, Russia).

After incubation, each agar plate was divided into 6–8 sectors, and one sector was randomly selected. All colonies present within the selected sector were collected for subsequent purification and analysis, irrespective of their colony morphology. This approach was used to minimize potential selection bias and obtain a more representative subset of the cultivable colonies.

The purified isolates were stored at −80 °C in MRS broth supplemented with 15% (*v*/*v*) glycerol in an ultra-low-temperature freezer (METHER Biomedical Co., Ltd., Hefei, China) until further analysis.

### 2.3. MALDI-TOF MS Identification of Isolates

Single colonies of the isolated microorganisms were selected for identification by MALDI-TOF mass spectrometry. A single colony was applied directly onto the MALDI target plate and suspended in 1 µL of a saturated α-cyano-4-hydroxycinnamic acid (α-HCCA) matrix solution containing 50% acetonitrile and 2.5% trifluoroacetic acid (TFA), followed by air-drying at room temperature. The target plate was loaded into a Microflex LT MALDI-TOF mass spectrometer (Bruker Daltonics, Billerica, MA, USA), positioned, and calibrated using a bacterial test standard. Spectra were acquired automatically using 40 laser shots at a frequency of 60 Hz over a mass range of 2000–20,000 Da. Spectral analysis was performed using Bruker MALDI Biotyper software (v4.0), with a minimum score of 2.0 applied for species-level identification [18,19,24].

Interpretation of results: score ≥ 2.0—reliable identification at the species level; score 1.7–1.99—identification at the genus level; score < 1.7—inconclusive identification. Isolates with the status ‘No peaks found’ were further analysed by 16S rRNA sequencing [20].

### 2.4. Genomic DNA Extraction

Genomic DNA was isolated from pure cultures using the DNA-sorb-B kit (InterLabService, Moscow, Russia, Cat No. K1-2-100) according to the manufacturer’s instructions. Briefly, cells were lysed in 300 µL of lysis buffer for 5 min at 65 °C, and nucleic acids were bound to 25 µL of universal sorbent for 7 min at room temperature. The sorbent was pelleted by centrifugation (5000 rpm) and washed sequentially with Wash Solutions 1 and 2, with centrifugation at 5000–10,000 rpm between steps. After air-drying (65 °C, 5–10 min), DNA was eluted in 50 µL of TE buffer at 65 °C for 5 min and separated from the sorbent by a final centrifugation step (12,000 rpm, 1 min).

DNA quality and concentration were measured by spectrophotometry (NanoDrop 1000, Thermo Fisher Scientific, Madison, WI, USA) and fluorimetry (Qubit 4 Fluorometer, Invitrogen, Thermo Fisher Scientific, Singapore).

### 2.5. Amplification and Sequencing of the 16S rRNA Gene

Amplification of the 16S rRNA gene fragment was performed using the universal bacterial primers 8F (5′-AGAGTTTGATCCTGGCTCAG-3′) and 806R (5′-GGACTACCAGGGTATCTAAT-3′) [25]. The PCR reaction was carried out in a total volume of 25 µL, containing 5 ng of template genomic DNA, 1 U of Hot Start Taq DNA Polymerase (Biolabmix, Novosibirsk, Russia, Cat No. E-3005), 0.2 mM of each dNTP, 1× Taq Buffer with KCl (Thermo Fisher Scientific, Waltham, MA, USA, Lot No. 2693545), 2.5 mM MgCl_2_ (Thermo Fisher Scientific, Waltham, MA, USA, Lot No. 2783956), and 10 pmol of each primer.

The PCR amplification programme included an initial denaturation step at 95 °C for 3 min; 30 cycles of denaturation at 95 °C for 30 s, annealing at 55 °C for 40 s, and extension at 72 °C for 60 s; and a final extension step at 72 °C for 10 min. The reaction was performed using a Mastercycler X50a thermal cycler (Eppendorf SE, Hamburg, Germany).

Amplicons were visualised on a 1.5% agarose gel and purified using magnetic (IzoGel LLC, Pushchino, Russia, Lot 7) particles based on a PEG/NaCl system [20].

Sequencing was performed using the Sanger method (BigDye Terminator v3.1, Applied Biosystems, Vilnius, Lithuania, Cat No. 370714) on an ABI 3730xl analyser (Applied Biosystems, Carlsbad, CA, USA).

### 2.6. Bioinformatic Analysis of 16S rRNA

The 16S rRNA gene chromatograms were processed in DNASTAR Lasergene v6.1 (SeqMan Pro; DNASTAR Inc., Madison, WI, USA). Low-quality regions at the sequence ends were automatically trimmed using the software’s built-in quality-based trimming algorithm (medium stringency setting), and sequences were additionally visually inspected to confirm the absence of overlapping peaks, high background noise, or ambiguous base calls in informative regions. Contigs were assembled using quality-weighted majority-rule consensus calling (75% majority threshold). Taxonomic identification was performed by comparing the resulting consensus sequences against the NCBI Nucleotide (nt) database using BLAST v.2.17.0. with default search parameters. Isolates were assigned to a species when the sequence identity to the top BLAST hit was ≥99%.

Phylogenetic analysis was carried out in MEGA 12 using the Neighbour-Joining method with 1000 bootstrap repetitions [26,27,28,29].

### 2.7. Whole-Genome Sequencing

Five representative strains were selected for WGS. Genomic DNA was extracted using the QIAamp DNA Mini Kit (Qiagen, Hildon, Germany; REF: 51306), following the manufacturer’s protocol for Gram-positive bacteria. Briefly, bacterial cells were lysed with lysozyme (20 mg/mL, 37 °C, 30 min), followed by proteinase K digestion in Buffer AL at 56 °C for 30 min. DNA was bound to the QIAamp spin column, washed with Buffers AW1 and AW2, and eluted in 200 µL of Buffer AE. Centrifugation steps were performed at 6000× *g* (washing) and 20,000× *g* (final spin) [30].

Libraries were prepared using the Collibri ES DNA Library Prep Kit (Illumina-compatible, Vilnius, Lithuania, Lot No. 2997589) and sequenced on the Illumina MiSeq platform (2 × 300 bp). Sequencing on the Illumina MiSeq (San Diego, CA, USA) platform was characterised by high-quality metrics. The cluster density was 1,327,000/mm^2^, and the proportion of bases with a quality score of Q30 or higher reached 80.0%.

### 2.8. Sequencing Data Analysis and Genome Assembly

Read quality was assessed using FastQC v0.12.1. Filtering was performed using SeqTK v1.4 and Sickle v1.33 [31].

De novo genome assembly was performed in SPAdes v3.15.5 using the command ‘--careful -k auto’.

Assembly quality was assessed using QUAST v5.2.0 (N50, L50, number of contigs, genome size, GC content).

Taxonomic identification was performed by BLAST alignment against the NCBI RefSeq representative genomes database [32,33].

### 2.9. Statistical Analysis

The relative abundance of taxa was calculated as the proportion of each species relative to the total number of isolates. Descriptive statistical analysis and data processing were performed using Microsoft Excel 365 (Microsoft Corporation, Redmond, WA, USA).

## 3. Results

### 3.1. Isolation and Taxonomic Identification of LAB Isolates

A total of 102 dairy and milk samples collected from the Turkestan region of Kazakhstan (Figure 1) were analysed using culture-dependent methods. Overall, 315 bacterial isolates displaying colony morphology characteristic of LAB were recovered.

Representative isolates were subjected to preliminary identification using MALDI-TOF MS, followed by partial 16S rRNA gene sequencing for taxonomic confirmation. In total, 124 isolates were subjected to taxonomic identification. After excluding contaminated cultures, non-LAB isolates, and isolates with inconclusive identification results, 79 confirmed LAB isolates were retained for further analyses (Table 1).

Among the confirmed isolates, partial 16S rRNA gene sequencing identified eight LAB species belonging to four genera: *Leuconostoc*, *Lactococcus*, *Lentilactobacillus*, and *Lacticaseibacillus*.

*Leuconostoc mesenteroides* was the predominant species, accounting for 48 isolates (60.8%), followed by *Lactococcus lactis* with 19 isolates (24.1%). The remaining species occurred at considerably lower frequencies, including *Leuconostoc lactis* (5.1%), *Lentilactobacillus kefiri* (2.5%), *Lentilactobacillus hilgardii* (2.5%), *Lacticaseibacillus paracasei* (2.5%), *Leuconostoc falkenbergense* (1.3%), and *Lentilactobacillus diolivorans* (1.3%).

The composition of LAB communities differed among the analysed dairy products. Kumis yielded the highest number of confirmed LAB isolates (37 isolates) and exhibited the greatest species diversity, whereas raw camel milk contained 16 isolates representing five species. Raw mare milk yielded 13 isolates, camel milk cheese yielded eight isolates, and shubat yielded five isolates. *Leuconostoc mesenteroides* was detected in all product types and predominated particularly in fermented dairy products, whereas *Lactococcus lactis* showed a comparatively higher abundance in raw camel milk. The remaining LAB species were recovered only sporadically and were represented by one to four isolates.

The distribution of LAB species across the analysed dairy products is presented in Figure 2. The stacked bars show the relative composition of the confirmed LAB isolates identified by 16S rRNA gene sequencing. *Leuconostoc mesenteroides* represented the largest proportion of isolates across the sample collection, with its predominance particularly pronounced in kumis.

Overall, the LAB composition varied among raw milk samples and fermented dairy products. Kumis was characterised by the highest species diversity and a pronounced predominance of *Leuconostoc mesenteroides*, whereas raw camel milk showed a comparatively higher proportion of *Lactococcus lactis*. Camel milk cheese and shubat exhibited lower species diversity, with *L. mesenteroides* remaining the predominant species. These findings demonstrate differences in the composition of cultivable LAB among the analysed sample types.

### 3.2. Phylogenetic Analysis of Isolates (16S rRNA)

Neighbour-Joining phylogenetic analysis based on partial 16S rRNA gene sequences supported the taxonomic assignment of the representative LAB isolates identified in this study (Figure 3). The phylogenetic analysis grouped the isolates into four genera, namely *Leuconostoc*, *Lactococcus*, *Lentilactobacillus*, and *Lacticaseibacillus*, consistent with the taxonomic assignments obtained from the sequence analysis.

Within the *Leuconostoc* tree (Figure 3A), the majority of isolates clustered with the reference strain *Leuconostoc mesenteroides*, whereas isolates LPG-L-174, LPG-L-199, LPG-L-235, and LPG-L-264 formed a separate cluster with *Leuconostoc lactis*. Isolate LPG-L-290 clustered with *Leuconostoc falkenbergense*, confirming its species-level identification.

The *Lactococcus* phylogenetic tree (Figure 3B) showed that all representative *Lactococcus lactis* isolates formed a highly supported monophyletic cluster with the corresponding reference strain.

The *Lentilactobacillus* phylogenetic tree (Figure 3C) separated the representative isolates into three species-level clusters corresponding to *Lentilactobacillus kefiri*, *L. hilgardii*, and *L. diolivorans*, each clustering with the respective type strain.

Similarly, isolates LPG-L-207 and LPG-L-305 grouped with the reference strain *Lacticaseibacillus paracasei* in the *Lacticaseibacillus* phylogenetic tree (Figure 3D), clearly separating them from other members of the genus.

### 3.3. Whole-Genome Sequencing and Genome Characterisation

Five representative isolates corresponding to the dominant and less abundant LAB species identified in this study were selected for whole-genome sequencing: LPG-L-32 (*Lactococcus lactis*), LPG-L-123 (*Leuconostoc mesenteroides*), LPG-L-148 (*Lentilactobacillus kefiri*), LPG-L-174 (*Leuconostoc lactis*), and LPG-L-290 (*Leuconostoc falkenbergense*).

Illumina MiSeq sequencing generated between 1.27 and 1.87 million reads per isolate (Table S2). The average read length ranged from 262 to 290 bp, while GC content of the sequencing reads varied from 34% to 42%. De novo genome assembly produced draft genomes ranging from 1.68 to 2.69 Mb in size. The circular genome maps of the five isolates, including the distribution of coding sequences (CDSs) on the forward and reverse strands, rRNA and tRNA genes, GC content, and GC skew, are presented in Figure 4. The smallest draft genome was obtained for LPG-L-174 (*Leuconostoc lactis*) at 1.68 Mb, whereas LPG-L-32 (*Lactococcus lactis*) had the largest genome at 2.69 Mb. The remaining genomes ranged from 2.02 to 2.59 Mb.

Initial taxonomic assignment based on BLAST v.2.17.0. analysis against the NCBI RefSeq database identified all five sequenced isolates at the species level. Strain LPG-L-32 was assigned to *Lactococcus lactis* subsp. *lactis*; LPG-L-123 to *Leuconostoc mesenteroides* subsp. *mesenteroides*; LPG-L-148 to *Lentilactobacillus kefiri*; LPG-L-174 to *Leuconostoc lactis*; and LPG-L-290 to *Leuconostoc falkenbergense*.

The OrthoANI analysis provided additional genome-level support for these assignments (Figure 5). Each isolate showed its highest OrthoANI similarity to reference genomes belonging to the corresponding species. LPG-L-32 showed a maximum OrthoANI value of 97.98% with *L. lactis*, while LPG-L-123 reached 99.22% with *L. mesenteroides*. LPG-L-148 exhibited the highest value of 99.47% with *L. kefiri*. For LPG-L-174 and LPG-L-290, the maximum OrthoANI values with conspecific reference genomes were 97.13% and 98.22%, respectively.

Comparisons with genomes representing other species generally yielded lower OrthoANI values, resulting in distinct genomic clustering of the sequenced isolates with their respective taxonomic groups (Figure 5).

Overall, the whole-genome assignments were consistent with the species-level identifications obtained previously using partial 16S rRNA gene sequences, while providing substantially stronger genome-wide evidence for taxonomic resolution of the selected isolates.

## 4. Discussion

A total of 315 bacterial isolates were initially recovered from the analysed samples. Of these, 124 representative isolates were selected for taxonomic identification, and 79 were ultimately confirmed as LAB (Supplementary Table S1). The remaining isolates were assigned to other bacterial groups or could not be reliably assigned at the required taxonomic level.

These findings highlight the limitations of the methods used for the preliminary recovery and taxonomic characterisation of LAB isolates. Although colony morphology and culture on MRS agar are useful for the preliminary recovery of presumptive LAB, they do not provide sufficient specificity for reliable taxonomic identification, as acid-tolerant non-LAB may also be recovered under these conditions [3,4,5,34,35]. Accordingly, only 79 of the 124 representative isolates were confirmed as LAB, while the remaining isolates were excluded because they represented other bacterial groups, contaminated cultures, or could not be reliably assigned at the required taxonomic level (Supplementary Table S1). Similar discrepancies between presumptive and confirmed LAB isolates have been reported in studies of traditional fermented dairy products [3,11,36]. These findings emphasize the need to combine preliminary culture-based isolation with molecular and genomic approaches for more reliable taxonomic characterisation [20,36,37].

MALDI-TOF MS and 16S rRNA gene sequencing provided complementary taxonomic information but also showed important limitations. MALDI-TOF MS enabled rapid screening and taxonomic assignment of a substantial proportion of isolates; however, some cultures did not yield suitable protein spectra, and one isolate (LPG-L-158) showed a discrepant result compared with 16S rRNA sequencing (Supplementary Table S1). Such limitations may be particularly relevant for food-associated microorganisms that are insufficiently represented in commercial reference databases [18,19,20,24], while rare LAB species or strains may also produce weak or variable spectra that do not allow for reliable matching [19,20]. Notably, 16S rRNA gene sequencing supported the classification of the confirmed isolates into eight LAB species belonging to four genera (Figure 2), but its relatively high sequence conservation limits its ability to discriminate among closely related species and provide sufficient resolution for definitive strain-level identification [20,38,39,40]. Phylogenetic analysis of the 16S rRNA sequences provided additional support for the observed taxonomic affiliations (Figure 3), whereas whole-genome sequencing of five representative isolates provided higher-resolution genomic evidence for their species-level assignments. Therefore, the taxonomic results obtained in this study should be interpreted according to the resolution and limitations of each method, with WGS providing the highest taxonomic resolution among the approaches applied in this study [20,21,36,37,38,39,40,41,42].

One of the most important findings of this study was the frequent recovery of *Leuconostoc mesenteroides* among the LAB isolates obtained from traditional dairy products from the studied region. According to the data in Figure 2, this species accounted for 60.8% of all confirmed LAB isolates and was detected among isolates recovered from all product types studied, including kumis, shubat, raw mare’s and camel’s milk, and camel’s milk cheese. The highest number of *L. mesenteroides* isolates was obtained from kumis, where 28 out of 37 confirmed LAB isolates were identified as this species (Figure 2). These findings indicate that *L. mesenteroides* was frequently recovered under the cultivation conditions applied in the present study and may represent an important cultivable LAB associated with the investigated traditional dairy products.

The results are consistent with previous studies, in which *L. mesenteroides* has been reported among the frequently detected microorganisms during the early stages of spontaneous milk fermentation [3,6,11,43]. The physiological characteristics of this species may contribute to its ability to grow under fermentation conditions. As an obligately heterofermentative lactic acid bacterium, *L. mesenteroides* can utilise a broad range of carbohydrates, producing lactic acid, carbon dioxide, acetate, or ethanol, as well as a variety of volatile compounds that may contribute to the sensory characteristics of fermented dairy products [6,7,8,34,44,45,46]. In addition, many strains are able to produce exopolysaccharides, which can contribute to texture and may improve bacterial tolerance to stresses encountered during fermentation [45,46]. These characteristics may contribute to the successful recovery of *L. mesenteroides* from fermented dairy products.

A direct comparison with the sequencing-based study of Tang et al. [47] provides further context for the cultivable LAB recovered in the present study. Tang et al. identified 119 bacterial species in koumiss using single-molecule real-time sequencing, with *Lactobacillus helveticus*, *Lactobacillus kefiranofaciens*, *Lactococcus lactis*, *Lactococcus raffinolactis*, and *Citrobacter freundii* among the dominant taxa. Of the eight LAB species identified in the present study, three—*Leuconostoc mesenteroides*, *Lactococcus lactis*, and *Lentilactobacillus kefiri* (reported as *Lactobacillus kefiri* by Tang et al.)—were also detected in that study, whereas the other five species were not detected. Notably, *L. mesenteroides* accounted for 60.8% of the confirmed LAB isolates in the present culture-dependent collection, compared with only 0.53% in the sequence-based profile reported by Tang et al. [47]. Similarly, *L. kefiri* represented only 0.82% in that study. These differences highlight the complementary nature of culture-dependent and culture-independent approaches. Sequence-based profiling provides a broader view of the microbial community, whereas culture-dependent methods selectively recover microorganisms capable of growing under the applied cultivation conditions. Therefore, the high recovery frequency of *L. mesenteroides* in the present study should be interpreted as a feature of the cultivable LAB fraction rather than as evidence of its predominance in the overall microbiota of the investigated dairy products.

The second most common species was *Lactococcus lactis*, accounting for 24.1% of the confirmed isolates. Unlike *L. mesenteroides*, this species was predominantly recovered from raw camel’s milk, where its proportion among the recovered LAB isolates was higher than in the other products studied, as shown in Figure 2. *L. lactis* is one of the most important members of starter culture microbiota in the dairy industry due to its high rate of lactose fermentation, intensive acidification of the medium, and its ability to produce bacteriocins and other antimicrobial compounds that can inhibit the growth of competing microorganisms [4,6,7,8,44,48,49]. The relatively frequent recovery of this species from camel’s milk may be associated with the physicochemical characteristics of this substrate.

Camel milk differs significantly from mare’s milk in terms of protein content, mineral composition, buffer capacity, and the concentration of natural antimicrobial components, including lactoferrin, lysozyme, immunoglobulins, and components of the lactoperoxidase system [11,12,13,14,50,51]. These factors can influence the growth and recovery of individual bacterial taxa under particular environmental conditions. Our findings are consistent with previously published reports indicating that the composition of raw milk can influence the microorganisms recovered from traditional fermented products of Central Asia [11,12,13,14,44,51,52,53].

The relatively high diversity of LAB identified in kumis is likely due to a combination of several factors. Mare’s milk is characterised by a high lactose content, which serves as an accessible source of carbon for lactic acid bacteria and promotes their growth [11,14]. Furthermore, the traditional method of preparing kumis involves the repeated addition of a portion of the previously fermented product (back-slopping), which ensures a constant influx of microorganisms from previous batches and sustains complex processes of microbial succession [11,14,47,54]. These practices may contribute to the relatively high diversity of cultivable LAB observed in kumis in the present study (Figure 2), although culture-based methods capture only a fraction of the total microbial community.

Conversely, the relatively low diversity of cultured LAB in shubat may be associated with the pronounced natural antimicrobial properties of camel milk. The presence of lactoferrin, lysozyme, and other biologically active components may limit the growth of certain groups of microorganisms during the early stages of fermentation, resulting in a less diverse cultivable LAB population [11,12,55]. Thus, the differences identified between kumis and shubat may reflect the combined influence of the biochemical characteristics of the raw milk and traditional production practices on the microorganisms recovered under the applied cultivation conditions.

Despite their relatively low prevalence, the detection of *Leuconostoc lactis*, *Leuconostoc falkenbergense*, *Lentilactobacillus kefiri*, *Lentilactobacillus hilgardii*, *Lentilactobacillus diolivorans*, and *Lacticaseibacillus paracasei* broadens the current knowledge of the diversity of cultivable LAB in traditional dairy products of Kazakhstan (Figure 2). Most of these species have previously been reported in fermented milk products, kefir grains, and other fermented food ecosystems, where they contribute to microbial succession, production of organic acids and aroma compounds, and maintenance of stable microbial communities during fermentation [3,11,56,57,58]. In contrast, *Leuconostoc falkenbergense* remains poorly characterised because of its relatively recent description, making its detection in traditional dairy products noteworthy and deserving of further ecological investigation [59]. Although these species were detected at low abundance, they demonstrate the taxonomic diversity of the cultivable LAB fraction and suggest that traditional fermented dairy products may represent a valuable reservoir of both dominant and less frequently recovered LAB [3,53,60].

Particular attention should be paid to the isolation of *Leuconostoc falkenbergense*, which was detected in only one kumis sample (Figure 2). Since its recent description, this species has been reported only rarely, and its occurrence in traditional fermented dairy products remains poorly documented, making its detection in kumis of particular ecological interest [59]. Its detection in a traditional product from Kazakhstan highlights the lack of research into the indigenous microbiota of fermented dairy products in Central Asia and points to the potential for further studies aimed at investigating the physiological, technological, and probiotic properties of this species.

The results of the phylogenetic analysis based on partial 16S rRNA gene sequences provided additional support for the taxonomic affiliation of the representative LAB isolates. As shown in Figure 3, the isolates were grouped according to their taxonomic affiliations within four genera: *Leuconostoc*, *Lactococcus*, *Lentilactobacillus*, and *Lacticaseibacillus*. Within the *Leuconostoc* lineage, a relatively large number of isolates, particularly those identified as *L. mesenteroides*, clustered closely with the corresponding reference sequence and with one another, forming several closely related groups. Similarly, the representative *Lactococcus lactis* isolates formed a relatively compact cluster, whereas the two *Lacticaseibacillus paracasei* isolates were positioned close to the corresponding reference sequence. These clustering patterns indicate a high degree of sequence similarity and phylogenetic relatedness among several of the recovered isolates. However, the close clustering of isolates based on partial 16S rRNA gene sequences should not be interpreted as definitive evidence of clonality. The relatively limited resolution of the 16S rRNA marker at the strain level does not allow closely related isolates to be reliably distinguished as identical or distinct clones. Therefore, the observed clustering may indicate the repeated recovery of genetically similar strains under the applied cultivation conditions, while confirmation of clonality or strain-level relatedness would require higher-resolution approaches, such as whole-genome sequencing or comparative genomic analysis.

Whole-genome sequencing of five representative LAB isolates provided additional genome-level confirmation of their taxonomic assignments. The selected isolates represented both predominant and less frequently detected species and included *Lactococcus lactis* (LPG-L-32), *Leuconostoc mesenteroides* (LPG-L-123), *Lentilactobacillus kefiri* (LPG-L-148), *Leuconostoc lactis* (LPG-L-174), and *Leuconostoc falkenbergense* (LPG-L-290). The resulting draft genome assemblies ranged from 1.68 to 2.69 Mb, with GC contents of 34.79–43.07% and N50 values of 42,030–118,665 bp. These genome characteristics were generally consistent with the reported genomic characteristics of the corresponding LAB species [38,41,42]. Differences in genome size, contig number, and assembly continuity were observed among the isolates, reflecting variation in the genomic characteristics and assembly properties of individual strains.

Taxonomic assignment based on comparison with the NCBI RefSeq database supported the species identities previously established using partial 16S rRNA gene sequencing. In addition, genome-level analysis enabled more detailed taxonomic resolution for selected isolates, with LPG-L-32 assigned to *Lactococcus lactis* subsp. *lactis* and LPG-L-123 to *Leuconostoc mesenteroides* subsp. *mesenteroides*. The OrthoANI analysis provided further support for these assignments (Figure 5). Each sequenced isolate showed its highest OrthoANI similarity to reference genomes belonging to the corresponding species, with values ranging from 97.13% to 99.47%. The distinct clustering of the isolates with conspecific reference genomes further supported their taxonomic placement.

The agreement between the whole-genome results, partial 16S rRNA gene sequencing, and phylogenetic analysis (Figure 3) demonstrates the value of combining complementary identification approaches. While 16S rRNA gene sequencing provided species-level identification for the broader collection of isolates, whole-genome comparison offered additional resolution for the five selected strains. However, because WGS was performed for only five of the 79 confirmed LAB isolates, these genome-level findings should be considered representative rather than indicative of the genomic diversity of the entire isolate collection.

Beyond taxonomic identification, the genome sequences generated in this study provide a basis for future investigation of genetic determinants potentially associated with technologically relevant traits, including carbohydrate metabolism, exopolysaccharide biosynthesis, bacteriocin production, stress tolerance, and other functional characteristics [30,61,62]. Nevertheless, the presence of genes associated with such traits would not by itself demonstrate their phenotypic expression. Functional predictions derived from genome sequences should therefore be complemented by physiological and experimental assays before strains are considered for technological or potential probiotic applications.

Several limitations should be considered when interpreting the findings of the present study. The culture-dependent isolation approach characterises only the cultivable fraction of the LAB population, while the sampling was restricted to the Turkestan Region and a single sampling period. In addition, the pH and temperature of the samples were not recorded at the time of collection. The pH values reported refer to the MRS cultivation medium, and incubation was carried out at a controlled temperature of 30 °C in a thermostat; these parameters do not reflect the original in situ conditions of the samples. These limitations should be considered in future studies involving broader sampling, culture-independent approaches, and direct measurement of physicochemical parameters at the time of sample collection.

Whole-genome sequencing of five representative LAB isolates provided higher-resolution genomic evidence for their taxonomic assignments. The selected isolates represented both frequently and less frequently recovered species, including *Lactococcus lactis* (LPG-L-32), *Leuconostoc mesenteroides* (LPG-L-123), *Lentilactobacillus kefiri* (LPG-L-148), *Leuconostoc lactis* (LPG-L-174), and *Leuconostoc falkenbergense* (LPG-L-290). The draft genomes differed in size and GC content, ranging from 1.68 to 2.69 Mb and from 34.79% to 43.07%, respectively, while OrthoANI values of 97.13–99.47% supported their assignment to the corresponding species. Genome-level analysis further resolved LPG-L-32 as *L. lactis* subsp. *lactis* and LPG-L-123 as *L. mesenteroides* subsp. *mesenteroides*. The circular genome maps (Figure 5) provide an overview of the distribution of coding sequences, rRNA and tRNA genes, GC content, and GC skew in the five isolates. Although the present WGS analysis was primarily intended for taxonomic characterisation, strain-specific genes, unique genomic regions, and functional genomic determinants were not systematically investigated. Therefore, no conclusions regarding unique functional features of the five isolates can be drawn from the present data, and such features would require comparative genomic and functional analyses.

## 5. Conclusions

This study characterised the cultivable lactic acid bacteria (LAB) recovered from traditional dairy products and raw milk from the Turkestan Region of Kazakhstan using a culture-dependent workflow complemented by MALDI-TOF MS, partial 16S rRNA gene sequencing, phylogenetic analysis, and whole-genome sequencing. Among 315 bacterial isolates initially recovered, 79 were confirmed as LAB and assigned to eight species representing four genera: *Leuconostoc*, *Lactococcus*, *Lentilactobacillus*, and *Lacticaseibacillus*.

*Leuconostoc mesenteroides* was the most frequently recovered species, accounting for 60.8% of the confirmed LAB isolates, followed by *Lactococcus lactis* (24.1%). The composition of the recovered LAB differed among the investigated product types, with *L. mesenteroides* frequently recovered from kumis and other investigated products, whereas *L. lactis* represented a substantial proportion of the LAB isolates recovered from raw camel’s milk. These patterns describe the cultivable fraction obtained under the applied isolation and incubation conditions and should not be interpreted as estimates of the relative abundance of LAB in the original microbial communities.

The detection of less frequently recovered species, including *Leuconostoc falkenbergense*, *Lentilactobacillus kefiri*, *L. hilgardii*, *L. diolivorans*, and *Lacticaseibacillus paracasei*, demonstrates the taxonomic diversity of the cultivable LAB fraction recovered from the investigated products. The detection of *L. falkenbergense* in kumis is particularly noteworthy given the limited information available on the occurrence of this species in traditional dairy ecosystems.

Whole-genome sequencing of five representative isolates provided genome-level support for their taxonomic assignments and yielded draft genomes ranging from 1.68 to 2.69 Mb with GC contents of 34.79–43.07%. OrthoANI values of 97.13–99.47% supported the corresponding species assignments, while genome-level analysis enabled subspecies-level resolution for selected isolates, including *Lactococcus lactis* subsp. *lactis* and *Leuconostoc mesenteroides* subsp. *mesenteroides*. Because WGS was performed for only five of the 79 confirmed LAB isolates, these genomic results should be considered representative of selected isolates rather than of the genomic diversity of the entire collection.

Overall, this study establishes a culture-based collection of indigenous LAB recovered from raw milk and traditional dairy products of the Turkestan Region and provides genome sequences for selected representatives. The isolates and genomic resources generated here provide a basis for subsequent comparative genomic, physiological, and technological studies. Further experimental characterisation is required to determine the functional and technological properties of individual isolates and to assess their suitability for potential starter culture or other applications.

## Figures and Tables

**Figure 1:**
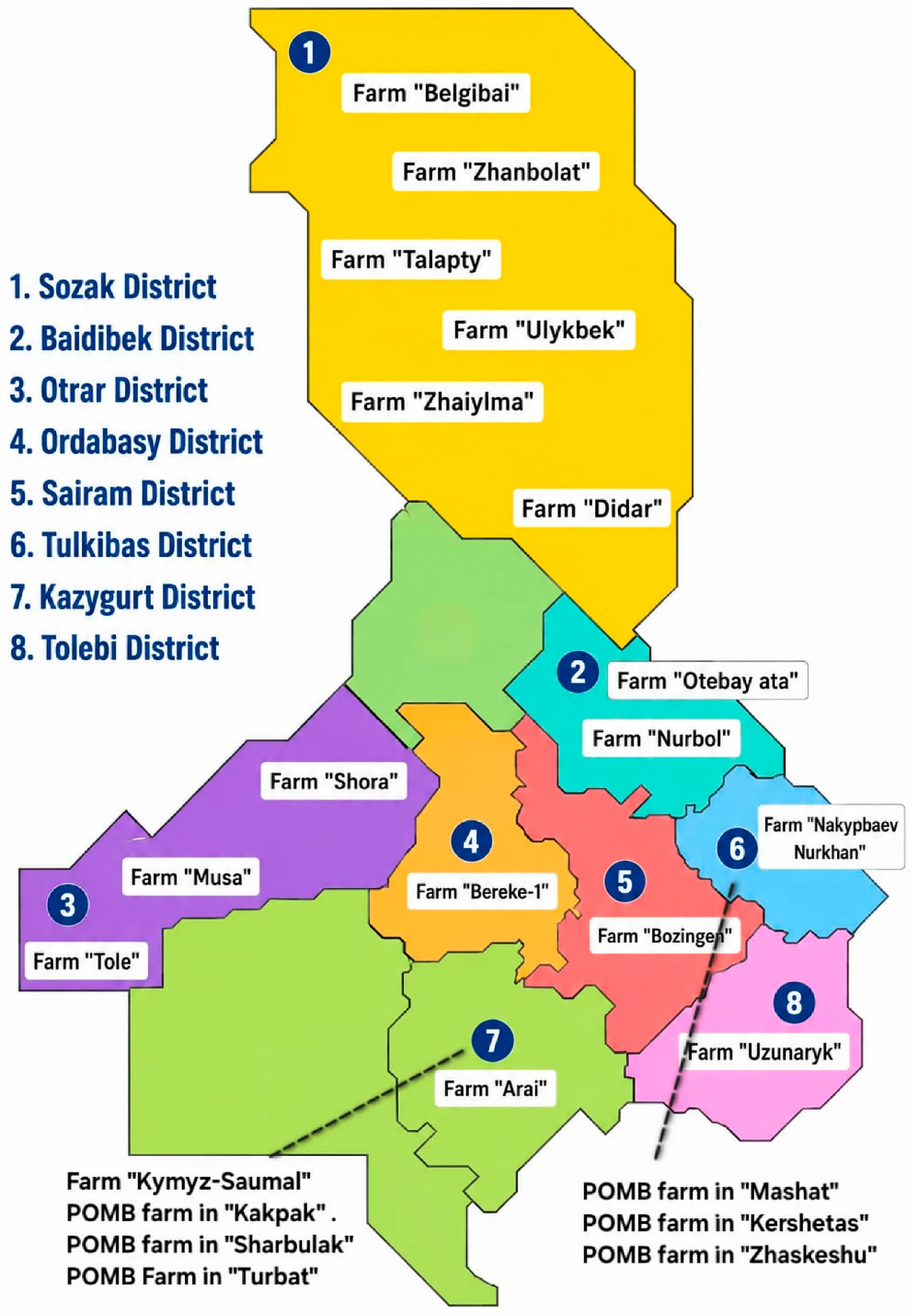
Geographic distribution of sampling sites in the Turkestan Region, Kazakhstan, showing the locations where camel’s milk, mare’s milk, shubat, kumis, and camel’s milk cheese samples were collected.

**Figure 2:**
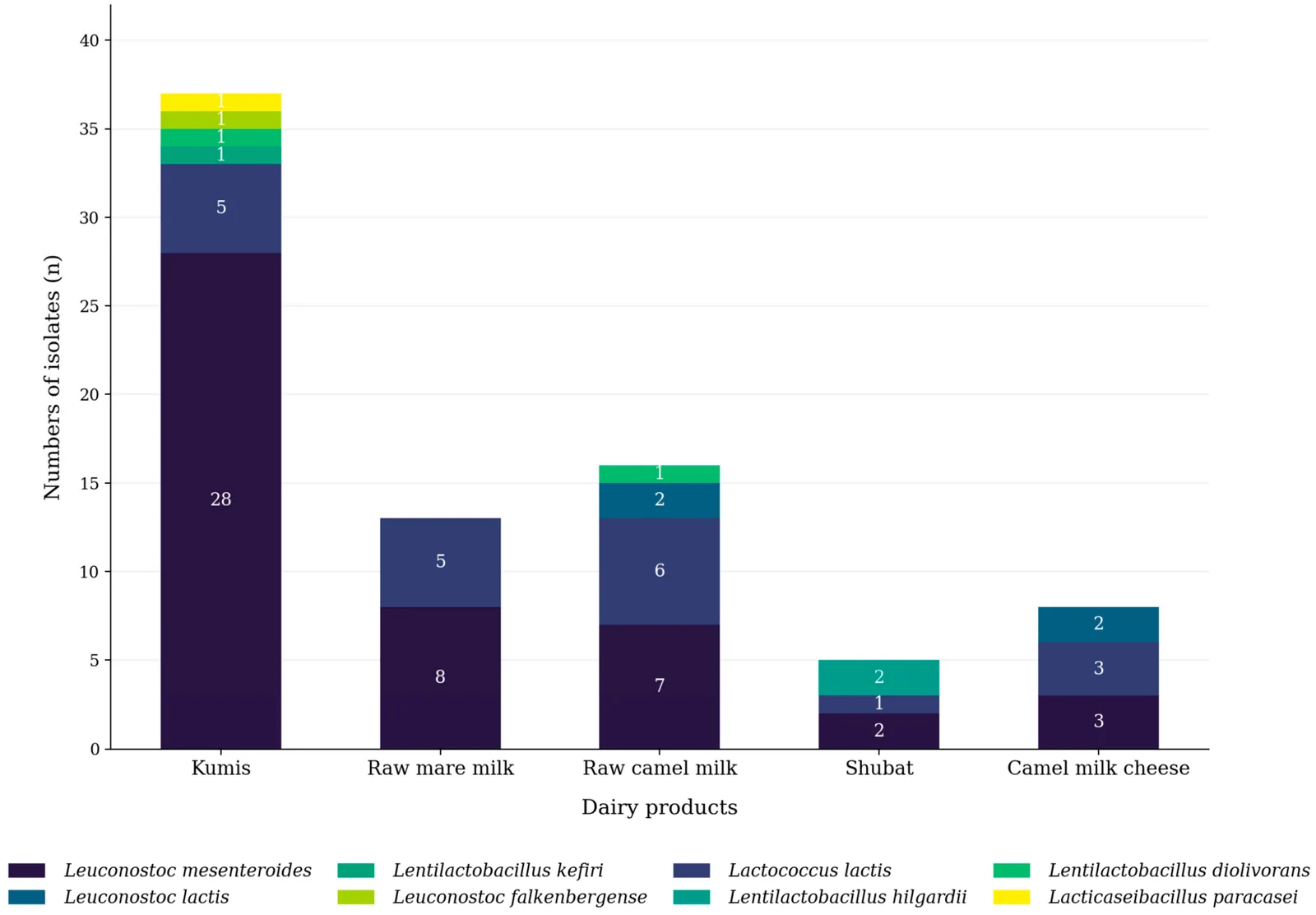
Distribution of confirmed LAB species among analysed raw milk and fermented dairy samples. Stacked bars show the relative composition of 79 LAB isolates identified by 16S rRNA gene sequencing.

**Figure 3:**
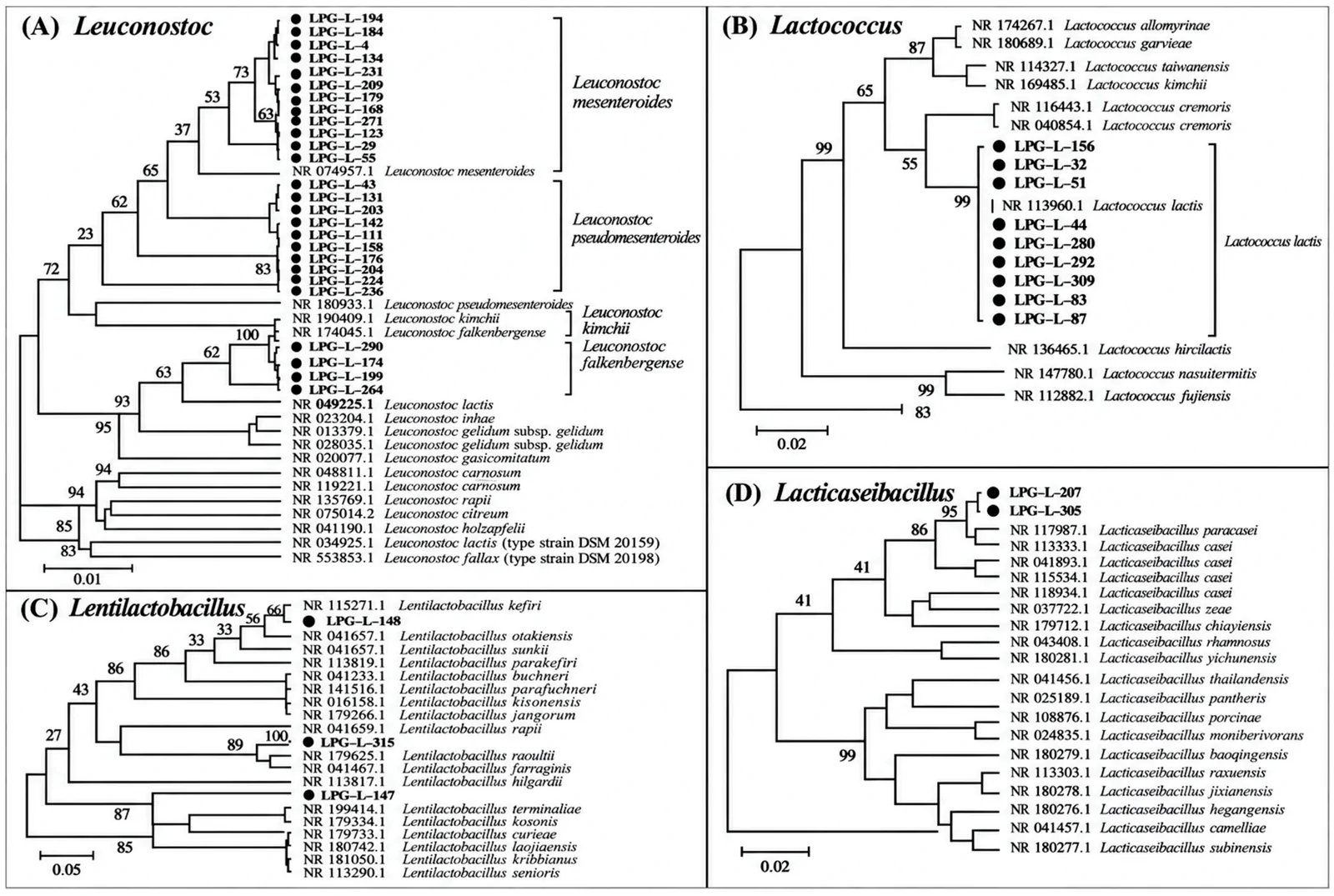
Neighbour-Joining phylogenetic trees based on partial 16S rRNA gene sequences showing the relationships between representative LAB isolates recovered from traditional dairy products and reference strains retrieved from GenBank. Bootstrap values (>50%) based on 1000 replicates are indicated at branch nodes. The scale bar represents nucleotide substitutions per site. Panels: (**A**) *Leuconostoc*, (**B**) *Lactococcus*, (**C**) *Lentilactobacillus*, and (**D**) *Lacticaseibacillus*.

**Figure 4:**
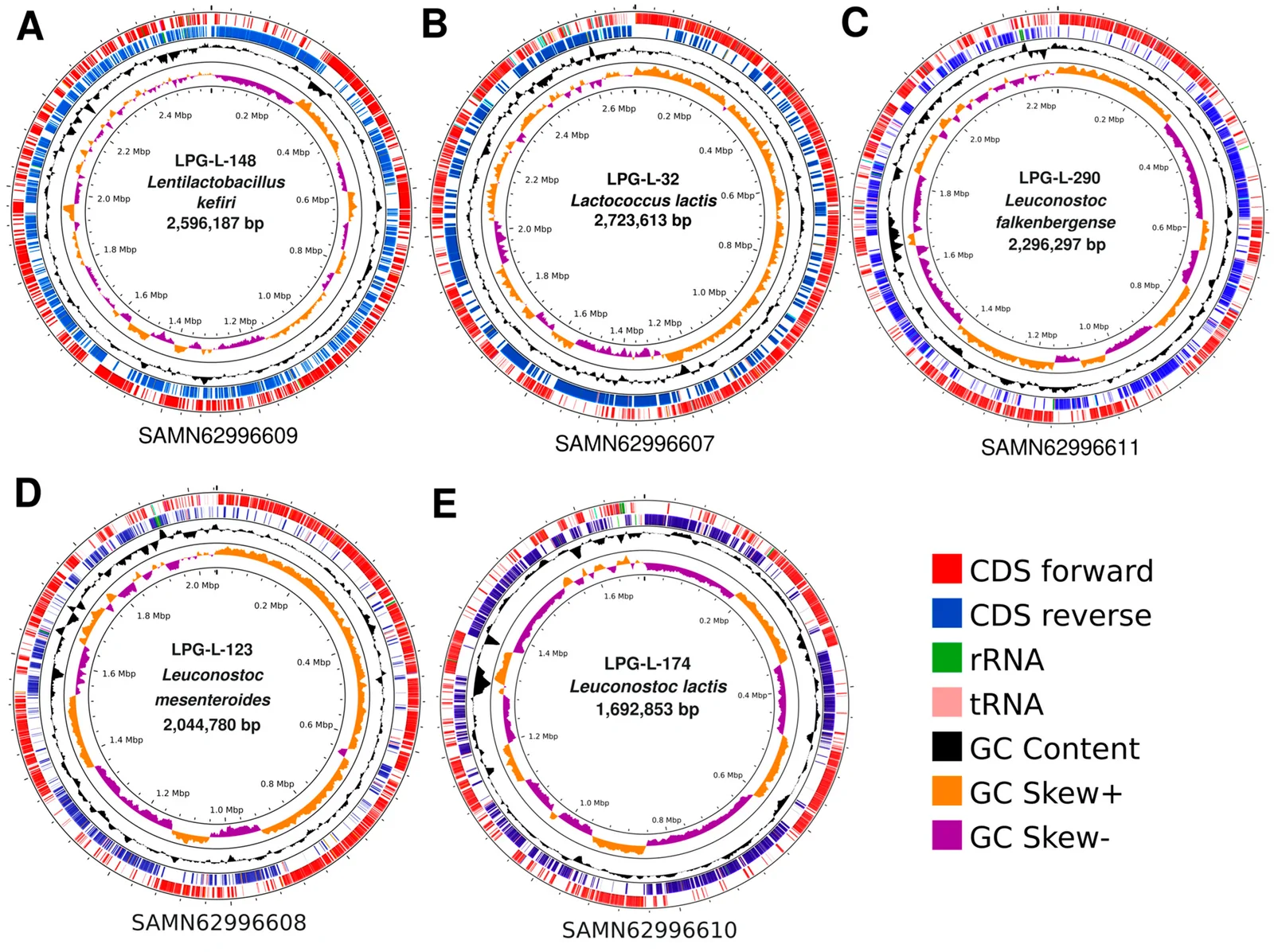
Circular genome maps of studied LAB isolates. The maps show coding sequences (CDSs) located on the forward and reverse strands, rRNA and tRNA genes, GC content, and GC skew. Panels correspond to (**A**) LPG-L-32, *Lactococcus lactis*; (**B**) LPG-L-123, *Leuconostoc mesenteroides*; (**C**) LPG-L-148, *Lentilactobacillus kefiri*; (**D**) LPG-L-174, *Leuconostoc lactis*; and (**E**) LPG-L-290, *Leuconostoc falkenbergense*. Genome size and taxonomic assignment are indicated within each map.

**Figure 5:**
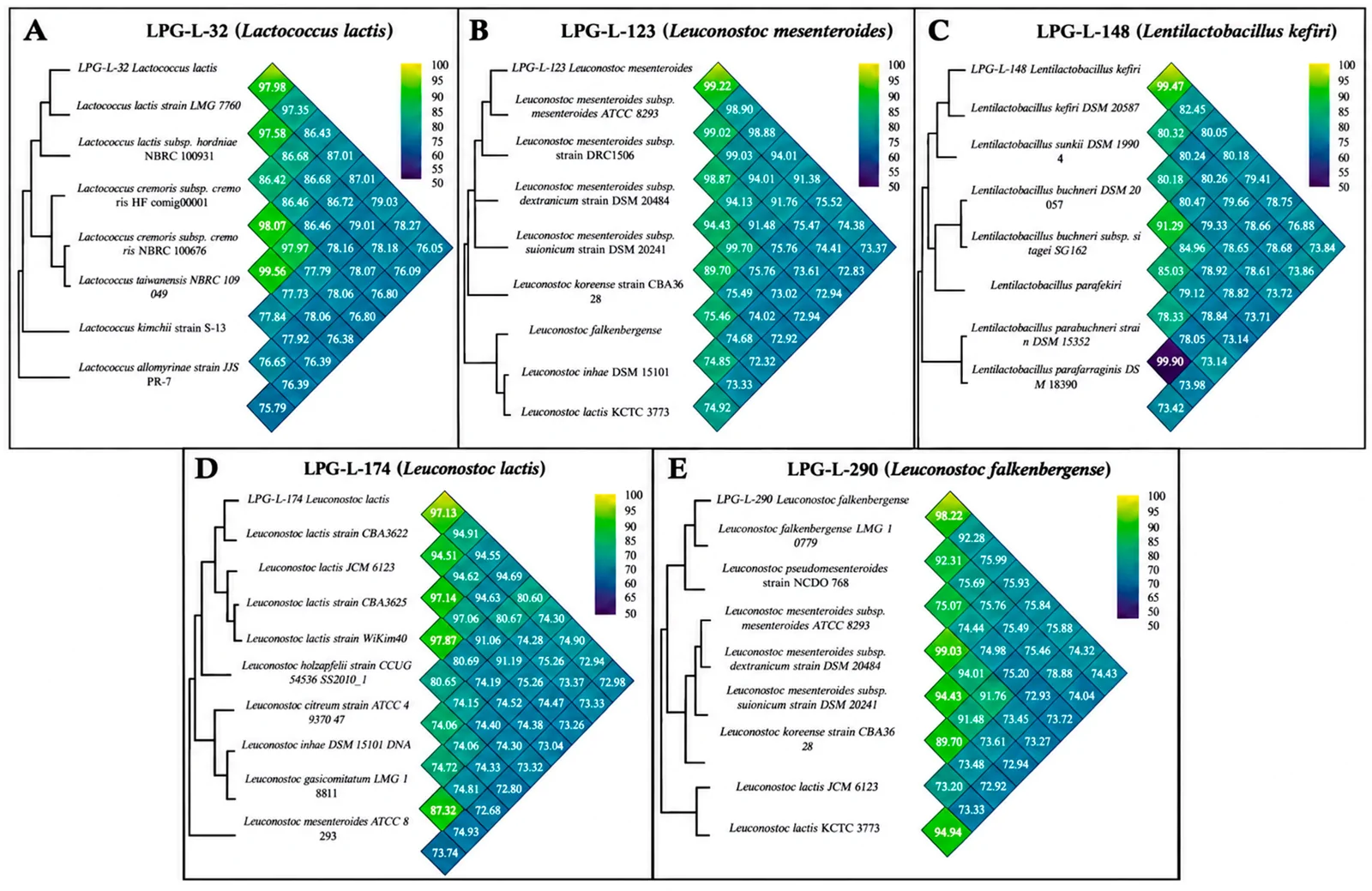
Genome-wide taxonomic assessment of five representative LAB isolates based on OrthoANI analysis: (**A**) LPG-L-32 (*Lactococcus lactis*), (**B**) LPG-L-123 (*Leuconostoc mesenteroides*), (**C**) LPG-L-148 (*Lentilactobacillus kefiri*), (**D**) LPG-L-174 (*Leuconostoc lactis*), and (**E**) LPG-L-290 (*Leuconostoc falkenbergense*).

**Table 1:** Outcome of bacterial identification.

Identification Outcome	Number of Isolates
Total isolates subjected to MALDI-TOF MS and 16S rRNA identification	124
Confirmed LAB isolates	79
Non-LAB isolates	12
Contaminated cultures	18
No reliable identification	6

## Data Availability

The original contributions presented in this study are included in the article/Supplementary Materials. Further inquiries can be directed to the corresponding author.

## Supplementary Materials

The following supporting information can be downloaded at: https://www.mdpi.com/article/10.3390/microorganisms14102201/s1, Table S1. Detailed identification of LAB isolates in the studied products. Table S2. Illumina MiSeq sequencing statistics. Table S3. (fragment). GenBank accession numbers for the whole-genome sequences of representative LAB strains.

## Abbreviations

The following abbreviations are used in this manuscript:

16S rRNA gene	16S ribosomal RNA gene
bp	Base pair
DNA	Deoxyribonucleic Acid
GC	Guanine–Cytosine content
LAB	Lactic acid bacteria
MALDI-TOF MS	Matrix-assisted laser desorption/ionization time-of-flight mass spectrometry
MRS	de Man, Rogosa and Sharpe medium
N aCl	Sodium chloride
NCBI	National Center for Biotechnology Information
PEG	Polyethylene glycol
POMB	Privately owned mare-breeding farms
