# Supplementary material for: Isolation and Genome-Resolved Characterisation of Selected Indigenous Lactic Acid Bacteria from Traditional Fermented Dairy Products of Kazakhstan
Source: Microorganisms. 2026 Oct 1;14(10):2201. doi: 10.3390/microorganisms14102201 (PMC13707972; doi:10.3390/microorganisms14102201)
Supplement: Supplementary file 1 [file microorganisms-14-02201-s001.zip › microorganisms-4528715-supplementary.pdf]

Table S1. Detailed identification of LAB isolates in the studied products.

No	Collection name	Product	MALDI-TOF results	16S rRNA results	16S rRNA sequence identity (%)	GenBank Accession Number (BLAST result)	Accession Number
1	LPG-L-4	Shubat	*Leuconostoc mesenteroides*	*Leuconostoc mesenteroides*	100	OQ848170.1	PZ951250
2	LPG-L-32	Raw camel milk	*Lactococcus lactis*	*Lactococcus lactis*	100	OP198571.1	PZ951251
3	LPG-L-35	Shubat	*Leuconostoc mesenteroides*	*Leuconostoc mesenteroides*	99,67	KF697618.1	PZ951252
4	LPG-L-43	Kumis	*Leuconostoc mesenteroides*	*Leuconostoc mesenteroides*	99,41	KF697618.1	PZ951253
5	LPG-L-44	Kumis	*Lactococcus lactis*	*Lactococcus lactis*	99,25	OK326486.1	PZ951254
6	LPG-L-51	Camel milk cheese	*Lactococcus lactis*	*Lactococcus lactis*	100	CP015904.2	PZ951255
7	LPG-L-59	Kumis	*Leuconostoc mesenteroides*	*Leuconostoc mesenteroides*	100	LC207998.1	PZ951256
8	LPG-L-83	Raw mare's milk	*Lactococcus lactis*	*Lactococcus lactis*	100	PV992514.1	PZ951257
9	LPG-L-87	Kumis	*Lactococcus lactis*	*Lactococcus lactis*	99,54	PV625125.1	PZ951258
10	LPG-L-105	Raw camel milk	no peaks found	*Lactococcus lactis*	100	OL336517	PZ951259
11	LPG-L-111	Raw mare's milk	*Leuconostoc mesenteroides*	*Leuconostoc mesenteroides*	100	PP916814	PZ951260
12	LPG-L-113	Shubat	*no peaks found*	*Lentilactobacillus kefiri*	100	CP115919	PZ951261
13	LPG-L-116	Raw mare's milk	*no peaks found*	*Leuconostoc mesenteroides*	100	PP916814	PZ951262
14	LPG-L-121	Raw camel milk	*no peaks found*	*Leuconostoc mesenteroides*	100	MW077401	PZ951263
15	LPG-L-123	Kumis	*Leuconostoc mesenteroides*	*Leuconostoc mesenteroides*	100	PP916814	PZ951264
16	LPG-L-127	Raw camel milk	no peaks found	*Leuconostoc mesenteroides*	100	MK659879	PZ951265
17	LPG-L-131	Raw camel milk	*Leuconostoc mesenteroides*	*Leuconostoc mesenteroides*	100	CP173687	PZ951266
18	LPG-L-134	Kumis	no peaks found	*Leuconostoc mesenteroides*	100	OQ850030	PZ951267
19	LPG-L-141	Kumis	no peaks found	*Leuconostoc mesenteroides*	100	OQ848079	PZ951268
20	LPG-L-142	Raw mare's milk	*Leuconostoc mesenteroides*	*Leuconostoc mesenteroides*	100	MW077401	PZ951269
21	LPG-L-147	Kumis	*Lentilactobacillus hilgardii*	*Lentilactobacillus hilgardii*	100	CP047121	PZ951270
22	LPG-L-148	Shubat	*Lentilactobacillus kefiri*	*Lentilactobacillus kefiri*	100	HM218364.1	PZ951271
23	LPG-L-149	Shubat	no peaks found	*Lentilactobacillus hilgardii*	100	CP047121	PZ951272
24	LPG-L-150	Camel milk cheese	no peaks found	*Leuconostoc mesenteroides*	100	MZ457325	PZ951273
25	LPG-L-154	Kumis	no peaks found	*Leuconostoc mesenteroides*	100	PP916814	PZ951274
26	LPG-L-155	Kumis	*Leuconostoc mesenteroides*	*Leuconostoc mesenteroides*	100	CP173687	PZ951275
27	LPG-L-156	Raw camel's milk	*Lactococcus lactis*	*Lactococcus lactis*	100	CP094474	PZ951276
28	LPG-L-157	Raw camel's milk	no peaks found	*Lactococcus lactis*	100	LC897780.1	PZ951277
29	LPG-L-158	Raw camel's milk	*Lactococcus lactis*	*Leuconostoc mesenteroides*	100	MZ457325	PZ951278
30	LPG-L-165	Kumis	*Leuconostoc mesenteroides*	*Leuconostoc mesenteroides*	100	MW077401	PZ951279
31	LPG-L-168	Kumis	*Leuconostoc mesenteroides*	*Leuconostoc mesenteroides*	100	MW077401	PZ951280
32	LPG-L-174	Camel milk cheese	no peaks found	*Leuconostoc lactis*	100	OM909138	PZ951281
33	LPG-L-175	Raw mare's milk	no peaks found	*Leuconostoc mesenteroides*	100	CP173687	PZ951282
34	LPG-L-176	Raw mare's milk	*Leuconostoc mesenteroides*	*Leuconostoc mesenteroides*	100	MW077401	PZ951283

35	LPG-L-179	Raw mare's milk	*Leuconostoc mesenteroides*	*Leuconostoc mesenteroides*	100	MZ457325	PZ951284
36	LPG-L-184	Camel milk cheese	*Leuconostoc mesenteroides*	*Leuconostoc mesenteroides*	100	MZ457325	PZ951285
37	LPG-L-188	Raw camel's milk	*no peaks found*	*Leuconostoc mesenteroides*	100	OR592654	PZ951286
38	LPG-L-192	Kumis	no peaks found	*Lactococcus lactis*	100	CP094474	PZ951287
39	LPG-L-194	Raw camel's milk	*Leuconostoc mesenteroides*	*Leuconostoc mesenteroides*	100	OM978567.1	PZ951288
40	LPG-L-196	Raw camel's milk	no peaks found	*Leuconostoc mesenteroides*	100	OQ848079	PZ951289
41	LPG-L-199	Camel milk cheese	*Leuconostoc lactis*	*Leuconostoc lactis*	100	CP157073	PZ951290
42	LPG-L-200	Camel milk cheese	no peaks found	*Leuconostoc lactis*	100	CP157073	PZ951291
43	LPG-L-203	Kumis	*Leuconostoc mesenteroides*	*Leuconostoc mesenteroides*	100	CP173687	PZ951292
44	LPG-L-204	Raw mare's milk	*Leuconostoc mesenteroides*	*Leuconostoc mesenteroides*	100	MZ457325	PZ951293
45	LPG-L-207	Kumis	*Lacticaseibacillus paracasei*	*Lacticaseibacillus paracasei*	100	CP146878	PZ951294
46	LPG-L-212	Raw mare's milk	no peaks found	*Lactococcus lactis*	100	CP094474	PZ951295
47	LPG-L-218	Kumis	no peaks found	*Leuconostoc mesenteroides*	100	CP173687	PZ951296
48	LPG-L-219	Kumis	*Leuconostoc mesenteroides*	*Leuconostoc mesenteroides*	100	MZ457325	PZ951297
49	LPG-L-222	Kumis	*Leuconostoc mesenteroides*	*Leuconostoc mesenteroides*	100	MW077401	PZ951298
50	LPG-L-226	Kumis	no peaks found	*Lactococcus lactis*	100	OL336517	PZ951299
51	LPG-L-227	Kumis	no peaks found	*Leuconostoc mesenteroides*	100	MZ457325	PZ951300
52	LPG-L-228	Raw camel's milk	no peaks found	*Lactococcus lactis*	100	AB830327	PZ951301
53	LPG-L-230	Kumis	no peaks found	*Leuconostoc mesenteroides*	100	PX470045	PZ951302
54	LPG-L-231	Kumis	*Leuconostoc mesenteroides*	*Leuconostoc mesenteroides*	100	MZ457325	PZ951303
55	LPG-L-232	Kumis	*Leuconostoc mesenteroides*	*Leuconostoc mesenteroides*	100	OQ848079	PZ951304
56	LPG-L-235	Raw camel's milk	*Leuconostoc lactis*	*Leuconostoc lactis*	100	CP157073	PZ951305
57	LPG-L-236	Kumis	*Leuconostoc mesenteroides*	*Leuconostoc mesenteroides*	100	MZ457325	PZ951306
58	LPG-L-237	Kumis	no peaks found	*Leuconostoc mesenteroides*	100	MZ457325	PZ951307
59	LPG-L-239	Kumis	no peaks found	*Leuconostoc mesenteroides*	100	CP173687	PZ951308
60	LPG-L-243	Kumis	no peaks found	*Leuconostoc mesenteroides*	100	CP173687	PZ951309
61	LPG-L-249	Kumis	no peaks found	*Leuconostoc mesenteroides*	100	MZ457325	PZ951310
62	LPG-L-250	Kumis	no peaks found	*Leuconostoc mesenteroides*	100	OQ848079	PZ951311
63	LPG-L-251	Kumis	no peaks found	*Leuconostoc mesenteroides*	100	CP173687	PZ951312
64	LPG-L-252	Kumis	*Leuconostoc mesenteroides*	*Leuconostoc mesenteroides*	100	CP173687	PZ951313
65	LPG-L-255	Kumis	no peaks found	*Leuconostoc mesenteroides*	100	PP916814	PZ951314
66	LPG-L-264	Raw camel's milk	*Leuconostoc lactis*	*Leuconostoc lactis*	100	OM909138	PZ951315
67	LPG-L-271	Raw mare's milk	*Leuconostoc mesenteroides*	*Leuconostoc mesenteroides*	100	MN809359	PZ951316
68	LPG-L-274	Kumis	*Lactococcus lactis*	*Lactococcus lactis*	100	CP094474	PZ951317
69	LPG-L-280	Raw mare's milk	*Lactococcus lactis*	*Lactococcus lactis*	100	OL336517	PZ951318
70	LPG-L-290	Kumis	*Leuconostoc pseudomesenteroides*	*Leuconostoc falkenbergense*	100	CP174386	PZ951319
71	LPG-L-291	Kumis	*Leuconostoc mesenteroides*	*Leuconostoc mesenteroides*	100	MK659879	PZ951320
72	LPG-L-292	Raw mare's milk	*Lactococcus lactis*	*Lactococcus lactis*	100	OL336517	PZ951321
73	LPG-L-299	Camel milk cheese	no peaks found	*Leuconostoc lactis*	100	PQ396392	PZ951322
74	LPG-L-301	Camel milk cheese	no peaks found	*Leuconostoc mesenteroides*	100	MT538644	PZ951323
75	LPG-L-305	Raw camel's milk	*Lacticaseibacillus paracasei*	*Lacticaseibacillus paracasei*	100	CP139083	PZ951324
76	LPG-L-309	Raw mare's milk	*Lactococcus lactis*	*Lactococcus lactis*	100	OL336517	PZ951325
77	LPG-L-311	Raw camel's milk	no peaks found	*Lactococcus lactis*	100	AB830327	PZ951326

78	LPG-L-314	Kumis	*Leuconostoc mesenteroides*	*Leuconostoc mesenteroides*	100	MW077401	PZ951327
79	LPG-L-315	Kumis	*Lentilactobacillus diolivorans*	*Lentilactobacillus diolivorans*	100	MT045520	PZ951328

Table S2. Illumina MiSeq sequencing statistics.

Parameter	Number of reads	Average read length, bp	Number of duplicates	GC%
LPG-L-32 (Lactococcus lactis)	1 316 234	285	11.6	34
LPG-L-123 (Leuconostoc mesenteroides)	1 361 176	290	20.9	36
LPG-L-148 (Lentilactobacillus kefiri)	1 268 784	287	13.5	40
LPG-L-174 (Leuconostoc lactis)	1 868 870	262	18.7	42
LPG-L-290 (Leuconostoc falkenbergense)	1 871 346	267	13.4	38

Table S3. (fragment). GenBank accession numbers for the whole-genome sequences of representative LAB strains.

GenBank Accession	Sample Name	Organism	TaxID	Strain
JCDNMH000000000	LPG-L-32	*Lactococcus lactis*	1358	LPG-L-32
JCDNMI000000000	LPG-L-123	*Leuconostoc mesenteroides*	1245	LPG-L-123
JCDNMJ000000000	LPG-L-148	*Lentilactobacillus kefiri*	33962	LPG-L-148
JCDNMK000000000	LPG-L-174	*Leuconostoc lactis*	1246	LPG-L-174
JCDNML000000000	LPG-L-290	*Leuconostoc falkenbergense*	2766470	LPG-L-290
